# A Phosphate-Regulated Promoter for Fine-Tuned and Reversible Overexpression in *Ostreococcus*: Application to Circadian Clock Functional Analysis

**DOI:** 10.1371/journal.pone.0028471

**Published:** 2011-12-12

**Authors:** El Batoul Djouani-Tahri, Frédéric Sanchez, Jean-Claude Lozano, François-Yves Bouget

**Affiliations:** 1 Universite Pierre et Marie Curie (Paris 06), Observatoire Océanologique, Banyuls/mer, France; 2 Centre national de la Recherche Scientifique, Unité Mixte de Recherche, UMR7621, LOMIC, Laboratoire d'océanographie microbienne, Banyuls/mer, France; Instituto de Biología Molecular y Celular de Plantas, Spain

## Abstract

**Background:**

The green picoalga *Ostreococcus tauri* (Prasinophyceae), which has been described as the smallest free-living eukaryotic organism, has minimal cellular ultra-structure and a very small genome. In recent years, *O. tauri* has emerged as a novel model organism for systems biology approaches that combine functional genomics and mathematical modeling, with a strong emphasis on light regulated processes and circadian clock. These approaches were made possible through the implementation of a minimal molecular toolbox for gene functional analysis including overexpression and knockdown strategies. We have previously shown that the promoter of the *High Affinity Phosphate Transporter* (*HAPT*) gene drives the expression of a luciferase reporter at high and constitutive levels under constant light.

**Methodology/Principal Findings:**

Here we report, using a luciferase reporter construct, that the *HAPT* promoter can be finely and reversibly tuned by modulating the level and nature of phosphate in culture medium. This *HAPT* regulation was additionally used to analyze the circadian clock gene *Time of Cab expression 1* (*TOC1*). The phenotype of a TOC1ox/CCA1:Luc line was reverted from arrhythmic to rhythmic simply by adding phosphate to the culture medium. Furthermore, since the time of phosphate injection had no effect on the phase of CCA1:Luc expression, this study suggests further that *TOC1* is a central clock gene in *Ostreococcus*.

**Conclusions/Perspectives:**

We have developed a phosphate-regulated expression system that allows fine gene function analysis in *Ostreococcus*. Recently, there has been a growing interest in microalgae as cell factories. This non-toxic phosphate-regulated system may prove useful in tuning protein expression levels quantitatively and temporally for biotechnological applications.

## Introduction

*Ostreococcus tauri* (Prasinophyceae) is one of the simplest free-living photosynthetic organisms described to date. This tiny eukaryotic green alga (around 1 µm in diameter) has minimal cellular organization, with only one chloroplast, a mitochondrion, a single Golgi body and no cell wall. Its small genome (12.6 Mbp in size) is extremely compact with very high gene density and an intergenic regions average of below 200 bp in size [1]. In addition, gene families and redundancy are extremely reduced. For example, several transcription factors (TFs) such as Basic Helix Loop Helix (BHLH), which are present at more than 150 copies per genome in higher plants, exist as single members in *Ostreococcus*, whilst *O. tauri* contains less than 200 TFs *in toto* [2]. In recent years, using functional genomics and system biology approaches, *O. tauri* has emerged as a promising model organism to study complex biological processes such as the circadian clock [3]–[7], the cell division cycle [8], [9] and starch biosynthesis pathways [10].

We have implemented tools for *in vivo* monitoring of gene expression using a luciferase reporter strategy as well as tools for gene functional analysis including overexpression and knockdown strategies in *O. tauri* [3], [9]. The pOtox overexpression vector relies on the promoter of the *PHO89/PHO4*-like *HAPT* gene [11], [12]. This promoter drives the expression of firefly luciferase at high and steady state levels under constant light in *O. tauri* [3]. Using this approach, we have functionally identified several genes involved in the circadian clock of *Ostreococcus*, including *Circadian Clock Associated 1* (CCA1), *TOC1* and putative photoreceptors with circadian clock function [3], [13], [14].

Circadian clocks rely in part on transcriptional translational feedback loops in which clock genes like *TOC1* activate the synthesis of their repressor (*CCA1*). Timely inducible overexpression systems provide an efficient means of circadian function analysis [15], [16]. For example, in *Neurospora*, step decreases in the concentration of the clock protein FRQ completely reset the phase of the clock consistent with FRQ being a central clock component [15].

A battery of inducible promoters is currently available for most model organisms, including plants and algae. They can be induced by specific exogenous molecules such as chemicals (alcohols, herbicides) and metabolites, or by environmental stimuli such as temperature or light [17]. For example, the heat shock-induced transcription promoter (HSP) *in Nicotiana tabacum* is rapidly and highly induced following temperature changes. In addition, its induction can be tuned by varying the temperature and the duration of the heat stimulus induction [18]. In *Pichia pastoris*, the promoter of *PHO89* has been used as an alternative inducible system to drive the expression of recombinant proteins for academic and industrial applications [19]. In the Prasinophyceae *Tetraselmis chui*, the *HAPT* gene was shown to be tightly regulated at the transcriptional level by inorganic phosphate [20].

In the present study we have investigated the regulation of the *HAPT* promoter by phosphate levels in *O. tauri* using a luciferase pHAPT:Luc construct. *HAPT* transcriptional activity was finely regulated by adjusting the phosphate concentration in the culture medium. Furthermore, this reversible overexpression system was tuned for fine functional analysis of the *TOC1* circadian clock gene.

## Results and Discussion

### Functional analysis of the *Ostreococcus tauri HAPT* promoter

**Figure 1:**
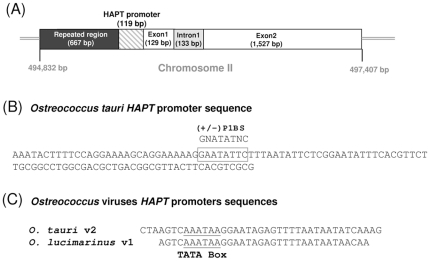
Map and nucleotide sequence of the *High Affinity Phosphate Transporter* promoters. (**A**) Scheme of the *O. tauri HAPT* gene. *HAPT* is localized on the atypical Chromosome II (ChrII) downstream of 667 bp-long repeated sequences. The gene structure (promoter, introns, exons) is supported by ESTs. (**B**) 119 bp-long *HAPT* putative promoter. A perfectly conserved Phosphate response element (P1BS) is located at position −76. (**C**) *HAPT* is one of the few genes conserved in *O. tauri* and *O. lucimarinus* viruses. The putative promoter sequences of these two viruses exhibit a conserved TATA Box at position −30 but no P1BS sequence.

The *HAPT* gene is located on the first half of the atypical chromosome II (ChrII) of *O. tauri* (Fig. 1A), which has a different GC% content compared to other chromosomes and has low synteny with *Ostreococcus lucimarinus* ChrII [21]. This part of ChrII contains introns with unusual splicing sequences and encompasses most of the genome repeated sequences and transposons [1]. The putative *HAPT* promoter sequence is only 119-bp long and contains at position −76 a GNATATNC PhR1-Binding Site (P1BS) found in the upstream regions of phosphate starvation responsive genes [22] (Fig. 1B). The *HAPT* gene was recently identified in the *O. tauri* Virus2 (OtV2) and *O. lucimarinus* Virus1 (OlV1) genomes suggesting that a lateral transfer has occurred [23]. The *HAPT* promoter sequences of both viruses are highly conserved (Fig. 1C). It is interesting that the P1BS motif was not detected in the 5′ regulatory sequences of OtV2 and OlV1, but instead a conserved putative TATA Box was found (Fig. 1C). This suggests that in the viruses the transcription of the *HAPT* gene may be constitutive rather than phosphate-controlled.

**Figure 2:**
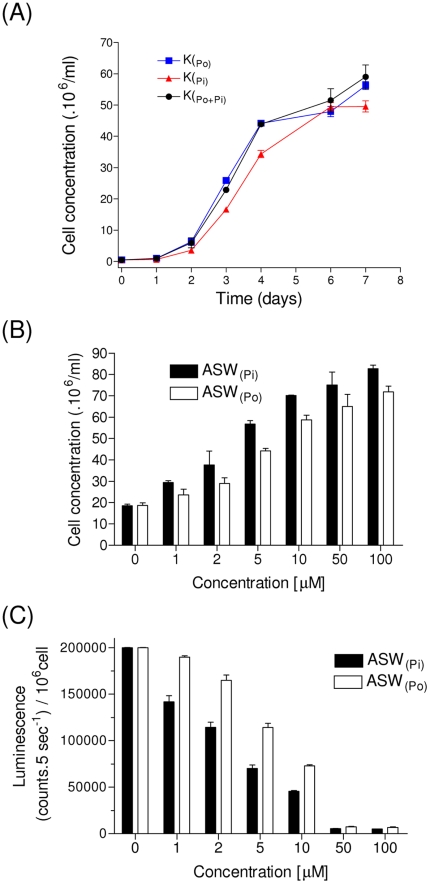
Effect of phosphate on cell growth and pHAPT:Luc promoter activity. (**A**) The effect of β-glycerophosphate (Po for organic phosphate), NaH_2_PO_4_ (Pi for inorganic phosphate) or combined Pi+Po was monitored on cell growth as recorded by flow cytometry. *Ostreococcus* cells were grown in Keller medium containing 10 µM β-glycerophosphate (K_Po_), Keller medium containing 10 µM NaH_2_PO_4_ instead of β-glycerophosphate (K_Pi_), and Keller Medium supplemented with 10 µM NaH_2_PO_4_K_Po+Pi_). (**B**) Cells were grown in ASW containing various concentrations of Pi or Po. Cell growth concentration was determined after 60 hours in culture. Similar dose response concentrations were obtained even though cell concentrations were slightly lower in ASW_(Pi)_ (N = 3, ±SD). (**C**) *In vitro* luminescence measurement of accumulated luciferase per cell over 60 hours in a pHAPT:Luc reporter line (N = 3, ±SD). A dose dependent inhibition was observed for both Po and Pi but the inhibition by Pi occurred at lower concentration.

To study the *HAPT* promoter regulation in *O. tauri*, we used a representative pHAPT:Luc reporter line, which is a transcriptional fusion between the *HAPT* promoter and the firefly luciferase [3]. *Ostreococcus* cells are usually cultivated in Keller medium that contains an organic source of phosphate (Po), provided as β-glycerophosphate (10 µM). Keller medium is based on natural sea water (NSW), supplemented with vitamins and nutriments (nitrate, β-glycerophosphate, trace metals), and therefore contains low levels of inorganic phosphate (between 0.1 and 100 nM), naturally present in the seawater. Cell growth was monitored in Keller medium containing different sources of phosphate: 10 µM β-glycerophosphate (K_Po_, standard Keller medium); 10 µM inorganic phosphate NaH_2_PO_4_ (K_Pi_) or 10 µMNaH_2_PO_4_+10 µM β-glycerophosphate (K_Po+Pi_). Similar growth curves were observed for the three different media conditions, suggesting that *O. tauri* cells assimilate organic or inorganic phosphate equally as well at these concentrations (Fig. 2A).

For the subsequent experiments, we developed an Artificial Sea Water (ASW) based on Keller medium, lacking Po and Pi (see Methods section). Cells were grown in ASW+10 µM Po (or Pi) to stationary phase before being diluted to a concentration of 10^6^ cells/ml in ASW containing various concentrations of Po (or Pi). Under low Po and Pi conditions, cell growth was strongly inhibited as measured after 60 hours (Fig. 2B). The luminescence measurement reflected the activity of total luciferase protein accumulated between 0 and 60 hours. The luminescence per cell was the highest for low phosphate concentrations (up to 40 fold between 0 and 50 µM [Pi]). This suggests that the *HAPT* promoter is strongly induced by phosphate starvation (Fig. 2C). Furthermore, a stronger inhibition was observed for Pi than for P_O_. Therefore, for subsequent experiments, inorganic phosphate was used to modulate the *HAPT* promoter activity.

### Kinetics of the *HAPT* promoter activity

**Figure 3:**
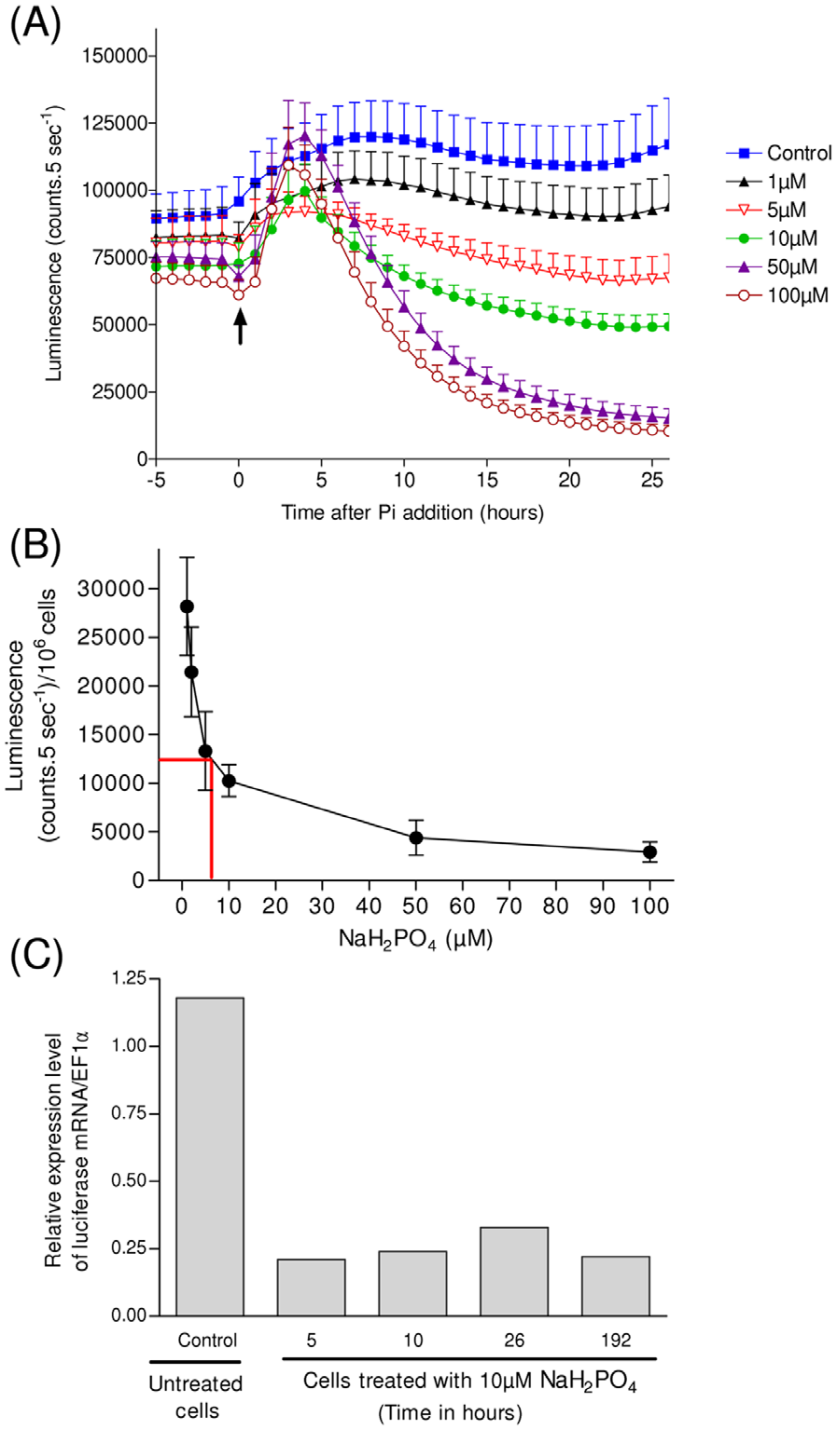
Time-course inhibition of *HAPT* promoter activity by inorganic phosphate. (**A**) *In vivo* kinetics of luciferase activity of pHAPT:Luc cells grown in ASW+10 µM Po before being exposed to various Pi concentrations for 28 hours from time 0. Maximal inhibition was observed for Pi concentrations above or equal to 50 µM (N = 3, ±SD). (**B**) Dose dependent inhibition of pHAPT:Luc *in vivo* luminescence by inorganic NaH_2_PO_4_. Luminescence measured 28 hours after Pi is reported as a function of Pi concentration. At this time a 50% inhibition is observed for [Pi]∼5 µM (N = 3, ±SD). (**C**) Corresponding luciferase mRNA levels measured at various times after Pi addition at a 50 µM concentration. Transcript amounts were quantified by real time quantitative RT-PCR and normalized to EF1α. Time 0 corresponds to the time of Pi addition. Control cells were grown in ASW 10 µM Po without Pi.

The kinetics of *HAPT* promoter activity were determined by adding various concentrations of Pi to pHAPT:Luc cells grown in the presence of luciferin (Fig. 3A). In these experimental conditions of continuous *in vivo* monitoring of luminescence, luciferase is inactivated after enzymatic reaction with luciferin. Consequently, the luminescence pattern reflects the dynamics of *HAPT* promoter activity more accurately than when measuring luciferase activity in extracts of cells grown without luciferin. For concentrations above or equal to 10 µM Pi, a transient increase in the pHAPT:Luc luminescence was observed during the first hours following Pi addition. Within 24 hours, the promoter activity dramatically decreased with increasing Pi concentrations. Twenty eight hours after Pi injection at concentrations above or equal to 50 µM, the residual pHAPT:Luc luminescence per cell was about six fold lower than in the control cells (grown in 10 µM Po without Pi) (Fig. 3B). At this time a 50 percent inhibition was observed for [Pi]∼5 µM. Quantitative analysis by real time RT-PCR showed that at [Pi] = 10 µM the luciferase transcript quickly dropped to a stable level (corresponding to about 20% of luciferase mRNA in control cells). This occurred as early as 5 hours after Pi addition (Fig. 3C). Differences were observed between the slow kinetics of pHAPT:Luc luciferase activity (Fig. 3A) and the fast decay of luciferase mRNA. These are likely to be due to the slow kinetics of luciferase inactivation upon oxidation of luciferin and/or to the luciferase stability. Data shown in Figure 3 indicate that *HAPT* transcriptional activity is strongly and quickly repressed upon phosphate addition. Concentrations above or equal to 10 µM [P_i_] appear to be a good compromise between the inhibition of p*HAPT* activity and cell growth.

### Activation of the *HAPT* promoter by phosphate starvation

**Figure 4:**
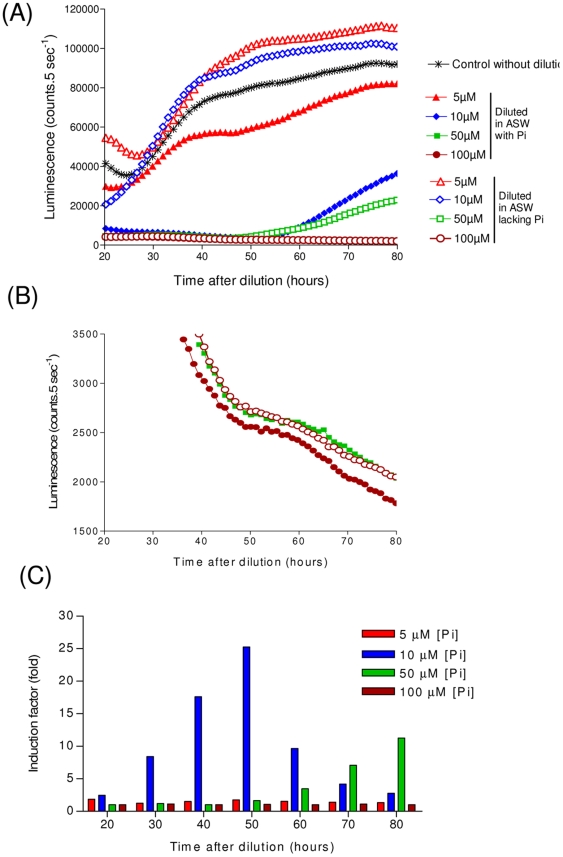
Activation of *HAPT* promoter by phosphate starvation. (**A**) Kinetics of pHAPT:Luc *in vivo* luciferase activity. Cells were grown to saturation in ASW containing various amount of Pi (5 to 100 µM) before being diluted 6 fold in ASW, either in the same medium (open symbols), or in phosphate free ASW to induce the *HAPT* promoter (filled symbols). Luminescence was recorded every hour from 0 (dilution time) to 80 hours. (B) Magnification of Y axis of the (A) graph to show the low luciferase values for high [Pi]. (**C**) The induction factor of pHAPT:Luc (luminescence in cells diluted in ASW without Pi/luminescence of cells diluted in medium with Pi) is reported as a function of the time after dilution. Control cells were grown in ASW 10 µM Po without Pi.

Ideally, a phosphate-tunable expression system should be capable not only of repressing, but also of up-regulating gene expression. In the next set of experiments, cells were grown to stationary phase in ASW medium supplemented with various concentrations of Pi between 5 and 100 µM. They were subsequently diluted 6 fold in ASW to reduce the initial Pi concentrations at time 0. Control cells were diluted in ASW containing 10 µM Po. For an initial concentration of 100 µM Pi, the *HAPT* promoter activity remained for at least 80 hours after dilution, most likely because the Pi concentration remained high (Fig. 4A and 4B). Cells grown in 50 µM [Pi] displayed a small increase in luminescence 60 hours after dilution, suggesting that upon Pi assimilation, Pi concentrations decreased to levels that are sufficient to activate the *HAPT* promoter.

The induction of *HAPT* transcriptional activity (luminescence of cells diluted in ASW lacking Pi/luminescence of control cells diluted in the initial culture medium) was calculated for each initial [Pi]. For a Pi concentration of 10 µM, a 25 fold induction of p*HAPT* activity was observed 50 hours after dilution (Fig. 4C). In these conditions, the luminescence increased at about 60 hours in control cells diluted with ASW containing Pi. This rise was likely the result of an assimilation of phosphate. At 5 µ M [Pi], only a moderate activation of pHAPT:Luc activity was observed in cells lacking Pi compared to those diluted in ASW containing 5 µM [Pi].

Taken together these results indicate that a six fold dilution of an initial [Pi] of 10 µM efficiently induces the activity of p*HAPT* within 40 hours. For longer inductions, the Pi concentrations should be adjusted upon phosphate assimilation in growing cells.

### Reversion of circadian overexpression phenotype by modulating the *HAPT* promoter activity

In gene functional analysis it is important to tune the expression level of genes of interest, without compromising cell growth. We have found that *Ostreococcus* cells grow equally well in both organic and inorganic phosphate. The addition of Pi at concentrations above 10 µM strongly inhibited the *HAPT* promoter, as demonstrated by luciferase activity and luciferase mRNA levels. Furthermore, the *HAPT* promoter could be modulated in a dose-dependent manner by adjusting the phosphate concentration in the culture medium.

**Figure 5:**
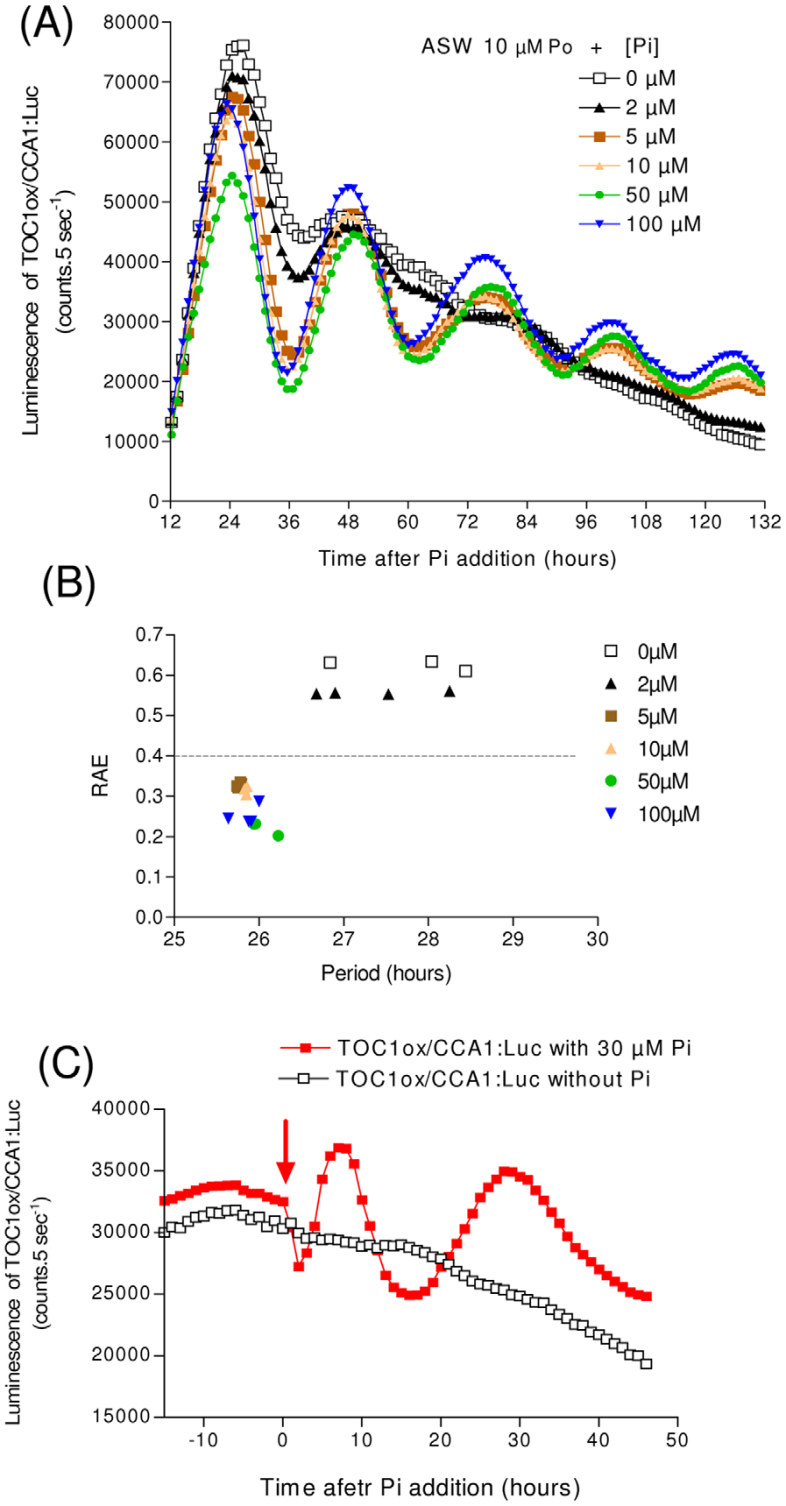
Reversion of circadian clock *TOC1*-overexpression phenotype by modulating the HAPT promoter activity. (**A**) TOC1ox/CCA1:Luc cells overexpressing *TOC1* under *HAPT* promoter display an arrhythmic profile of CCA1:Luc luminescence under constant light when grown in ASW containing 10 µM Po (standard Keller Medium). Pi was added at the time 0 of transfer to constant light (after an initial 6 hour dark pulse) to repress the HAPT promoter activity. For [Pi]≥5 µM, TOC1ox/CCA1:Luc cells recovered rhythmic expression of CCA1:Luc. (**B**) Quantitative effect of phosphate on TOC1ox/CCA1:Luc rhythmicity in constant light. To assess rhythmicity, relative amplitude error (RAE) estimation was performed over at least 3 days of recording, using the FFT-NLLS program from BRASS. For all [Pi]≥5 µM, robust rhythms were observed (RAE<0.4, N = 3), the period variations were below 1 hour and no dose-dependent effect was observed. (**C**) Addition of Pi to a 30 µM final concentration at time 98 restored a rhythmic pattern of CCA1:Luc in *TOC1* overexpressing line. Time 0 corresponds to time of Pi addition.

In the next experiment, we aimed to lower the level of overexpression in order to reverse strong arrhythmic circadian phenotypes. Circadian clocks rely largely on transcriptional oscillators which are based on negative feedback loops. Such an oscillator in *Ostreococcus* is formed by the CCA1/TOC1 couple, CCA1 repressing the transcription of *TOC1*. The TOC1 protein in turn activates the transcription of *CCA1* through an unknown mechanism. Clamping *TOC1* at high levels leads to arrhythmic CCA1 expression, consistent with *TOC1* being involved in the clock. We applied a range of [Pi] on a TOC1ox/CCA1:Luc arrhythmic line grown in ASW+10 µM [Po], to modulate the level of *TOC1* overexpression (Fig. 5A). Below 5 µM [Pi], the TOC1ox/CCA1:Luc were considered as arrhythmic in constant light even though one additional damped oscillation was observed at 2 µM [Pi], (Fig. 5A and 5B). For higher [Pi], the TOC1ox/CCA1:Luc recovered a rhythmic expression pattern of CCA1:Luc. Notably, when recovering the rhythmicity of the luciferase reporter for [Pi]≥5 µM, no significant period changes were observed between the different phosphate concentrations (*Pv* = 0.096 in an ANOVA test; Fig. 5A and 5B). This all-or-none response may be due to the extreme sensitivity of *Ostreococcus* rhythms to altering *TOC1* levels [3], [4]. The arrhythmic TOC1ox/CCA1:Luc line also quickly recovered rhythmicity upon addition of Pi to cells in the course of the experiment (Fig. 5C).

### Fine circadian clock function analysis

**Figure 6:**
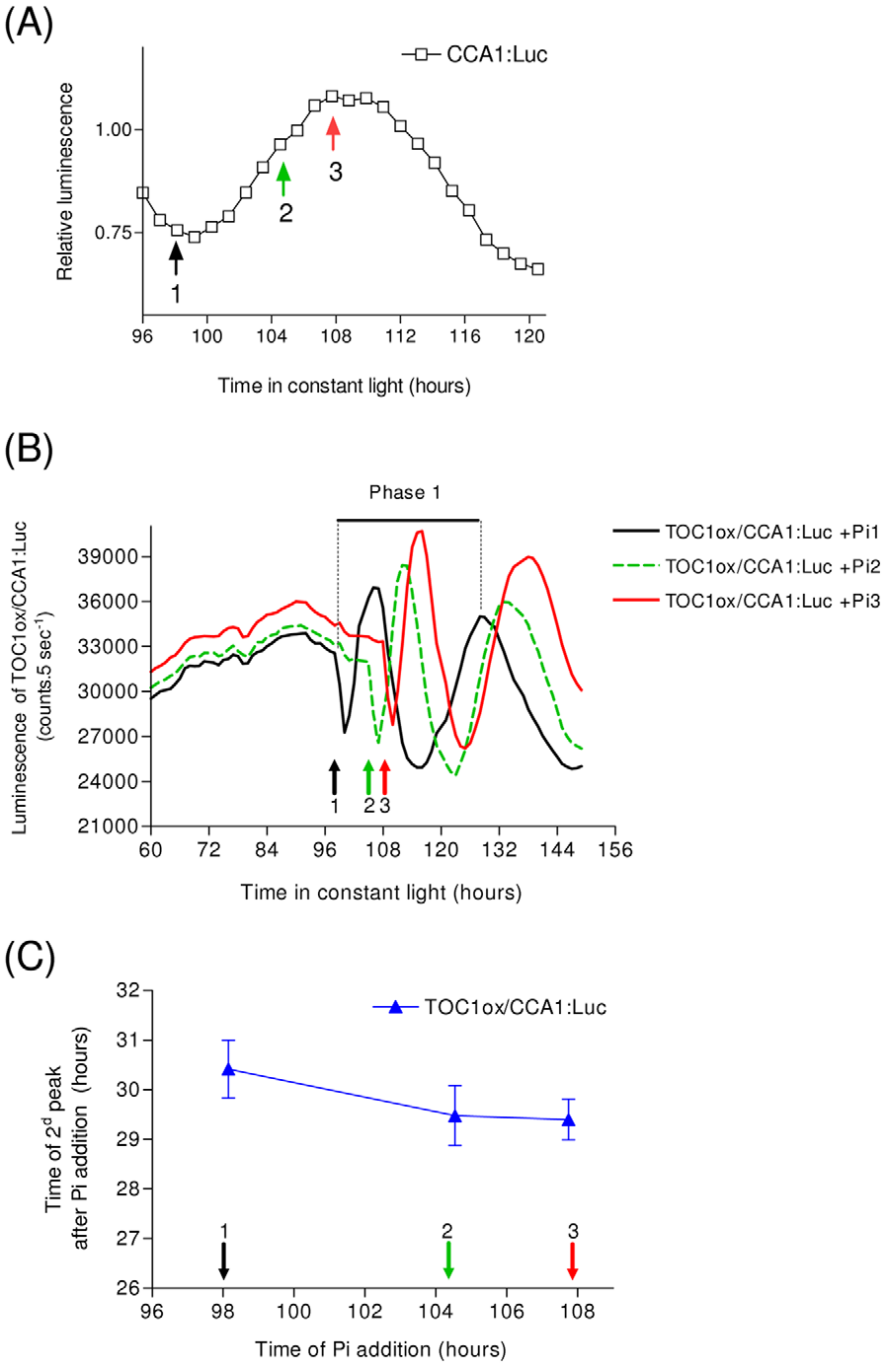
Analysis of *TOC1* circadian function. (**A**) Experimental protocol. Luciferase reporter lines CCA1:Luc and TOC1ox/CCA1:Luc were released in constant light at Time 0 after an initial synchronization by a 6 hour dark pulse. Pi was added at a 30 µM final concentration at three different times (t1 = 98, t2 = 104 and t3 = 108 hours). Arrows indicate the three times of Pi addition relative to the phases of CCA1:Luc control line (**B**). For the three times of Pi addition, a rhythmic expression pattern of CCA1:Luc was recovered. The phase of CCA1:Luc peak of expression relative to the time of Pi injection was determined on the second peak of CCA1:Luc (e.g. Phase 1 corresponds to the time t1 of Pi addition). (**C**) The phase of CCA1:Luc peak of expression is plotted as a function of the time of Pi addition. Note that for the three times, no circadian gating of CCA1:Luc response is observed (N = 3, ±SD).

An apparent arrhythmic phenotype must be carefully interpreted since a residual circadian clock function can persist in constant light, a phenomenon known as “masking” [15]. This could arise from saturation of photo-transduction input pathways under constant light [24]. We reasoned that if an underlying masked clock remained active in an apparently arrhythmic line, its sensitivity to changes in the *TOC1* levels should depend on the time of day. In this case, lowering *TOC1* overexpression (upon Pi addition) in the TOC1ox/CCA1:Luc line should have a different effect on circadian rhythms depending on the time of phosphate addition. In contrast, if *TOC1* is a central clock gene, the peak of expression of CCA1:Luc should occur at fixed times after Pi addition. To test this hypothesis, Pi (30 µM final concentration) was added at different times to a TOC1ox/CCA1:Luc line synchronized by an initial 6 hour dark pulse. This relatively high Pi concentration was chosen so that [Pi] would not drop to levels sufficient to activate the *HAPT* promoter upon phosphate assimilation in the course of the experiment. Times of Pi addition corresponded to different phases of the CCA1:Luc cycle, including peak and trough (Fig. 6A).

The arrhythmic TOC1ox/CCA1:Luc cells recovered rhythmicity quickly upon Pi addition (Fig. 6B). The peak of expression of CCA1:Luc was determined after one transitory oscillation and plotted as a function of the time of Pi addition (Fig. 6C). Data analysis showed that the peak phase was constant (29.81 h±0.69, N = 3), with no significant difference between the 3 times of Pi addition (*Pv* = 0.505 in an ANOVA test). These results indicate that there is no masked clock gating the CCA1:Luc response upon alteration of *TOC1* overexpression level. A similar approach developed in *Arabidopsi*s using an ethanol-induced expression system demonstrated that pulses of *TOC1* expression, due to a strong post-translational control, did not elicit phase shifts [16]. Our results in *Ostreococcus* suggest that lowering *TOC1* overexpression by repressing the *HAPT* promoter is sufficient to restore circadian rhythms of CCA1 indicating the importance of transcriptional regulations in the *Ostreococcus* circadian clock.

### Conclusions

We have demonstrated that the the *O. tauri HAPT* gene short promoter can be regulated by controlling the level of exogenous phosphate in the culture medium. At the transcript level changes occur within 5 hours of phosphate addition. Circadian phenotypes can be reversed by simply adding phosphate to cells and the central role of *TOC1* for clock function has been highlighted.

Compared to existing eukaryotic microalgal overexpression systems that are based on metal- inducible promoters such as copper or nickel [25], the *HAPT* promoter regulation relies on phosphate, an essential non-toxic nutriment, which usually becomes limiting when cells reach stationary phase. In the future, this phosphate-tunable system could be used to uncouple cell growth from recombinant protein expression. This would be particularly important in the development of *Ostreococcus* as a cell factory for biotechnological applications.

## Methods

### Algal culture and culture medium

*O. tauri* 0TTH95 WT strain and transgenic reporter lines were grown in flasks (Sarstedt) or white 96-well microplates (Nunc, Perkin Elmer) under constant light at a light intensity of 20 µmol quanta m^−2^ s^−1^. In the first experiment, cells were grown in standard Keller medium which contains natural seawater supplemented with trace metals and vitamins [8]. The effect of the phosphate source on cell growth was determined by adding organic β-glycero-phosphate or inorganic NaH_2_PO_4_ to a final concentration of 10 µM. For subsequent experiments, a Keller-based Artificial Sea Water (ASW) medium was developed. This modified Keller medium contains 24.55 g/l NaCl, 0.75 g/l KCl, 4.07 g/l MgCl_2_ 6H_2_O, 1.47 g/l CaCl_2_2H_2_O, 6.04 g/l MgSO_4_7H_2_O, 0.21 g/l NaHCO_3_. The concentration of phosphate was adjusted using organic β-glycerophosphate or inorganic NaH_2_PO_4_ depending on the experiment. For circadian experiments, 5 µl of NaH_2_PO_4_ (1.2 mM stock solution) was gently added to 200 µl of cell cultures grown in microplates. Cell counting was performed by flow cytometry using a Cell Lab Quanta™ SC MPL – (Beckman Coulter). Cells were fixed in 0.25% glutaraldehyde for 20 min before flow cytometry analysis.

### Monitoring and analysis of *in vivo* bioluminescence

*O. tauri* pHAPT:Luc, TOC1ox/CCA1:Luc and CCA1:Luc lines have been described elsewhere [3]. Cells were grown to saturation in microplates under constant light. Cells were plated at equal cell density (10.10^6^ cell/ml) in culture medium containing D-luciferin (10 µM). Cell synchronization was achieved by a 6 hour dark period before placing the cells under constant light. Luminescence was acquired for 5 sec every hour using an automated microplate luminometer (Berthold LB Centro). Statistical analyses of circadian rhythm were performed using BRASS software (Biological Rhythms Analysis Software System, P.E. Brown, Warwick University). FFT-NLLS analysis (Fast Fourier Transform NonLinear Last Square) was used to estimate Relative Amplitude Error (RAE) (a measure of goodness-fit to theoretical sine wave) and Free Running Period (FRP) that were taken as an objective measure of the rhythmicity of the bioluminescence traces [26]. Lines with RAE values above 0.4 displayed no detectable rhythms of luminescence and were considered arrhythmic (AR). The number of tested lines is represented by N. Error bars of standard deviation (SD) and are represented by ±SD. Statistical analyses were performed using ANOVA test, α = 5% (*Pvalue* is indicated).

### *In vitro* measurements of bioluminescence in cell extracts

Cells cultures grown to saturation (200 µl) were extracted in lysis buffer (100 mM potassium phosphate, 1 mM EDTA, 1 mM DTT, 1% triton X-100, and 10% glycerol, pH 7.8). Luciferase essays were performed in a luminometer (Berthold LB Centro) after injection of luciferase reagent (20 mM Tricine, 5 mM MgCl2, 0.1 mM EDTA, 3.3 mM DTT, 270 µM Coenzyme A, 500 µM luciferin, and 500 µM ATP, pH 7.8). The luminescence value was normalized to the total number of cells scored by flow cytometry.

### RNA extraction and quantitative RT–PCR analysis

RNA was extracted using the RNeasy-Plus Mini kit (Qiagen). Quantitative Real time RT-PCR was carried out on a LightCycler 1.5 (Roche Diagnostic, Mannhein, Germany) with LightCycler DNA Master SYBR Green I (Roche Molecular Biochemicals, Mannhein, Germany). Primers were designed with LightCycler Probe Design2 software (Roche Diagnostic, Mannhein, Germany). The following primers were used for qRT-PCR analysis of the luciferase mRNA: Forward primer: (5′- CGGAAAGACGATGACGG-3′), Reverse primer: (5′-GCCCTTCTTGGCCTTTATG -3′). The *O. tauri* elongation factor 1 α (EF1α) was used as internal reference. Results were analyzed using the comparative critical threshold (ΔΔ*C*T) method.
